# Karst vegetation coverage detection using UAV multispectral vegetation indices and machine learning algorithm

**DOI:** 10.1186/s13007-023-00982-7

**Published:** 2023-01-23

**Authors:** Wen Pan, Xiaoyu Wang, Yan Sun, Jia Wang, Yanjie Li, Sheng Li

**Affiliations:** 1 Research Institute of Subtropical Forestry, Chinese Academy of Forestry, No. 73, Daqiao Road, Fuyang, Hangzhou, 311400 Zhejiang China; 2 College of Forestry, Nanjing Forestry University, Nanjing, China; 3 Chun’an County Forestry Administration, Hangzhou, Zhejiang China

**Keywords:** UAV, Machine learning, Classification, Karst, Vegetation indices

## Abstract

**Background:**

Karst vegetation is of great significance for ecological restoration in karst areas. Vegetation Indices (VIs) are mainly related to plant yield which is helpful to understand the status of ecological restoration in karst areas. Recently, karst vegetation surveys have gradually shifted from field surveys to remote sensing-based methods. Coupled with the machine learning methods, the Unmanned Aerial Vehicle (UAV) multispectral remote sensing data can effectively improve the detection accuracy of vegetation and extract the important spectrum features.

**Results:**

In this study, UAV multispectral image data at flight altitudes of 100 m, 200 m, and 400 m were collected to be applied for vegetation detection in a karst area. The resulting ground resolutions of the 100 m, 200 m, and 400 m data are 5.29, 10.58, and 21.16 cm/pixel, respectively. Four machine learning models, including Random Forest (RF), Support Vector Machine (SVM), Gradient Boosting Machine (GBM), and Deep Learning (DL), were compared to test the performance of vegetation coverage detection. 5 spectral values (Red, Green, Blue, NIR, Red edge) and 16 VIs were selected to perform variable importance analysis on the best detection models. The results show that the best model for each flight altitude has the highest accuracy in detecting its training data (over 90%), and the GBM model constructed based on all data at all flight altitudes yields the best detection performance covering all data, with an overall accuracy of 95.66%. The variables that were significantly correlated and not correlated with the best model were the Modified Soil Adjusted Vegetation Index (MSAVI) and the Modified Anthocyanin Content Index (MACI), respectively. Finally, the best model was used to invert the complete UAV images at different flight altitudes.

**Conclusions:**

In general, the GBM_all model constructed based on UAV imaging with all flight altitudes was feasible to accurately detect karst vegetation coverage. The prediction models constructed based on data from different flight altitudes had a certain similarity in the distribution of vegetation index importance. Combined with the method of visual interpretation, the karst green vegetation predicted by the best model was in good agreement with the ground truth, and other land types including hay, rock, and soil were well predicted. This study provided a methodological reference for the detection of karst vegetation coverage in eastern China.

**Supplementary Information:**

The online version contains supplementary material available at 10.1186/s13007-023-00982-7.

## Background

Karst environments are areas where slightly dissolved rock outcrops and efficient acid hydrolysis create spectacular dissolved landforms [1]. Karst areas have been distributed around the world and cover about 15% of Earth’s surface [2]. The carbonate rocks in Southeast Asia, which is the largest karst area in the world, are continuously exposed and its ecological environment is extremely vulnerable to human activities [3]. Karst areas have high complexity and strong spatial and temporal heterogeneity. It is a comprehensive reflection of the staggered distribution of various ground objects such as bedrock, vegetation, and soil cover [4].

As an important part of global vegetation, karst vegetation not only provides a great carbon sink function but also provides a series of ecological services, which has always been a research hotspot in the field of global change [5]. The vegetation in karst areas is critical for maintaining fragile local ecosystems [6]. In addition, vegetation is a significant sensitive factor that reflects changes in the ecological environment of karst areas [7]. The coverage of dry vegetation such as litter and bare surface soil also plays an important role in the characterization and evaluation of the degree of land degradation [8]. Therefore, it is particularly important to figure out the classification and distribution of vegetation populations in karst areas.

In the early years, many domestic and foreign studies on vegetation coverage detection in karst areas introduced many methods. Early research mainly used fieldwork methods for vegetation coverage detection. For instance, Blasi et al. [9] conducted a field investigation and multivariate analysis of the vegetation communities in the karst tectonic basin of the Majella Massif called plant sociology methods. Bátori et al. [10] using the Moving Segmentation Window (MSW) technique, nested analysis, and Principal Coordinate Analysis (PCoA) conducted a field survey in southern and northern Hungary between 2005 and 2012 and revealed vegetation patterns in ocean trough. Although field surveys hold the advantages of high accuracy but time- and cost-consuming, and which are not suitable for the detection of vegetation in large-scale karst areas.

Recently, with the continuous improvement of the spatiotemporal and spectral resolution of remote sensing technology, massive remote sensing image datasets have emerged as the times require [11]. It is worth noting that the extraction of ground object information, especially vegetation information, employing remote sensing images has been largely studied in remote sensing research. In terms of multispectral, the overall accuracy of vegetation coverage detection was improved by 5.57% compared with traditional supervised classification using the combination of the Back Propagation Neural Network (BPNN) model and Landsat-8 Operational Land Imager (OLI) multispectral remote sensing images [12]. Regarding hyper-spectrum, through linear spectral separation and pixel separation methods, hyperspectral images can be used to extract ecological indicators such as karst vegetation fraction and vegetation abundance, which can characterize vegetation coverage to a certain extent for vegetation inversion [13, 14]. However, most remote sensing satellite images involve visual interpretation and computer-aided digital processing of aerial photography and satellite images, which are highly subjective and inefficient, and limit the ability to distinguish and identify ground objects in karst areas [15, 16].

Karst areas are characterized by thin soil layers and exposed rocks. Since the ground cover in karst areas is often a mixture of several types (vegetated and non-vegetated), it is difficult to accurately extract the main features of vegetation cover [17]. Vegetation, soil, and rocks have high reflectance to different wavelengths of visible light, which makes the difference between karst vegetation and non-vegetation in UAV multispectral images significant. Xiao et al. [18] pointed out the method of using vegetation index to distinguish karst vegetation from non-vegetation. To solve the problems of low image resolution and inaccurate vegetation coverage detection, UAV remote sensing technology is rapidly going mainstream [19]. Among recent innovations, UAVs are suitable for tracking and assessing vegetation conditions in time, with several advantages: (1) They can fly at low flight altitudes, providing high-definition aerial imagery and high spatial resolution, fine details of vegetation can be detected, (2) flights can be flexibly scheduled according to critical moments imposed by vegetation over time, (3) they can be acquired in different ways using different sensors and the range of perception systems of vegetation spectrum (visible, infrared, thermal), (4) this technique can also generate the Digital Surface Model (DSM) with Three-Dimensional (3D) vegetation measurements by using highly overlapping images and applying an image reconstruction procedure with Structure from motion (SFM) techniques [20]. As such, UAVs are a cost-effective tool for acquiring high spatial resolution 3D data of plants and trees where satellite platforms are not feasible, filling the gap between ground-based equipment and other traditional remote sensing systems. Beyond that, UAV-based digital imagery can effectively replace data collected through laborious, subjective, and destructive manual fieldwork [21]. Due to these advantages, the UAVs are becoming quite suitable platforms for vegetation coverage detection in karst areas [22], mainly using RGB [23, 24], multispectral [25, 26], hyperspectral [27], and lidar [28] sensors.

The large amount of detailed data embedded in UAV imagery requires the development and application of powerful advanced analysis programs for extracting information related to vegetation structure and biochemical composition to better understand relevant plant traits [29, 30]. Bolin Fu et al. [31] set the flight altitude of the UAV to 105 m uniformly and used an optimized Random Forest—Decision Tree (RF-DT) model to extract vegetation communities, which explored the optimal detection variables for various types of vegetation. Zhang et al. [32] set the flight altitude to 100 m and used UAV-based hyperspectral images, combined with SVM and Edge-Preserving Filter (EPF), to automatically extract tree canopies damaged by Chinese pine caterpillars and perform more refined classification. Mäyrä et al. [33] collected data from a flight altitude of 1500 m and compared the performance of Three-Dimensional Convolutional Neural Network (3D-CNN), GBM, DL, etc. in the classification of individual tree species in hyperspectral data and used the best performing 3D-CNN as a complete tree species map was generated for the study area. These studies show that machine learning algorithms such as RF, SVM, GBM, and DL algorithms have been widely used in the detection of vegetation coverage detection. When combining different UAV flight altitudes and feature variables, the detection accuracies of various models show significant differences.

At present, the vegetation cover research of karst areas is focused on the continuous karst area in southwest China, which is the center of the karst area in Southeast Asia [34]. There are obvious differences between the karst areas in Southwest and Eastern China. The former is difficult to grow due to the sparse soil cover and there are many tree species with strong stress resistance such as cypress. In contrast, the latter has deep soil cover, lush vegetation, and high coverage. In addition, there are few studies on vegetation cover in karst areas of eastern China. Therefore, it is necessary to understand the changes in vegetation cover in the karst areas of eastern China to achieve sustainable development of karst ecosystems.

In this study, we focus on karst ecosystems to demonstrate the use of efficient analytical methods for UAV multispectral datasets (such as RF, SVM, GBM, and DL) and aim to answer the following questions:

1. In the multispectral data of different UAV flight altitudes, how accurate are the four models of RF, SVM, GBM, and DL in identifying karst vegetation coverage?
2. In the optimal variable selection, how is the vegetation indices' importance distribution of the best models at each flight altitude?
3. Combined with the method of visual interpretation, how well do the vegetation distribution of the three flight altitudes predicted by the best model fit with the respective real vegetation distribution?

## Materials

### Study area

**Fig. 1:**
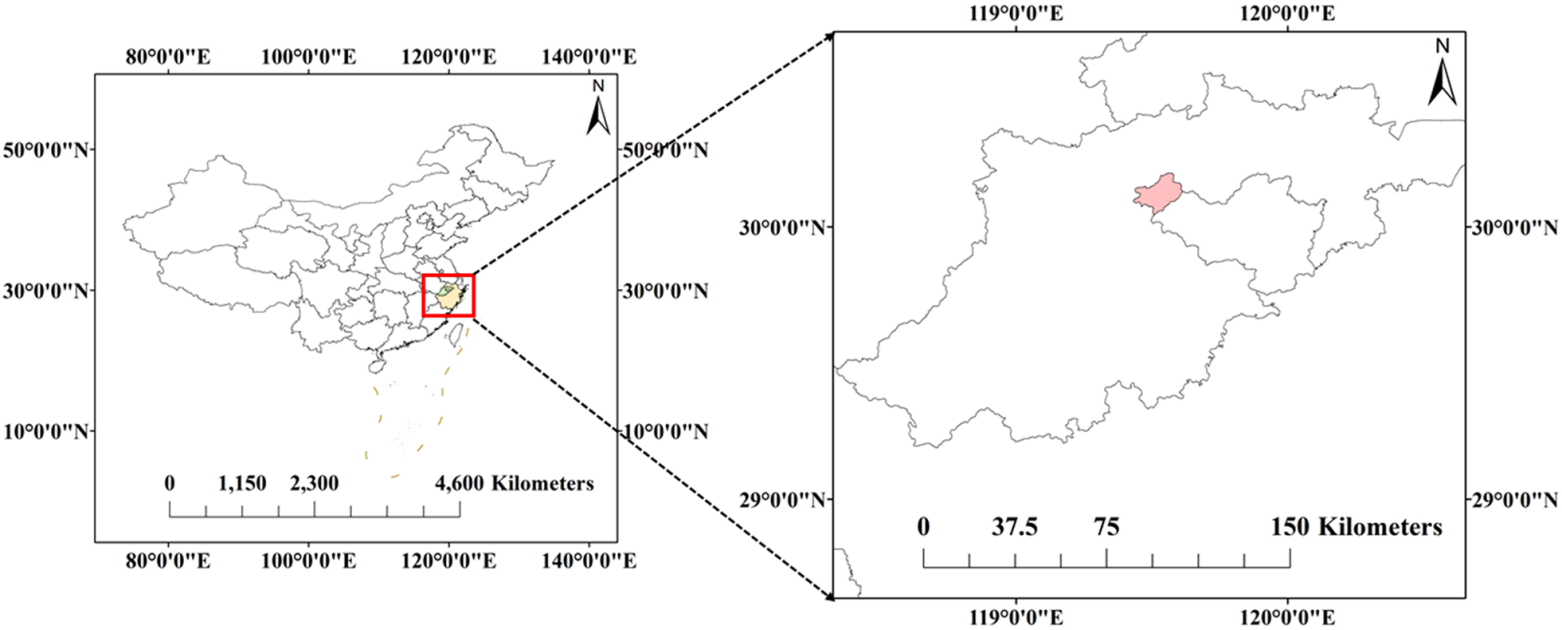
The sketch map of study area

The study area is located in Wanshi, Fuyang, Hangzhou, Zhejiang, China (30°6′9″N, 119°31′46″E; Fig. 1), with a total area of 157.9 square kilometers. The region has a subtropical monsoon climate, with an average annual temperature of about 16.3 °C and an average annual rainfall of about 1479.3 mm. The vegetation cover in the study area is mainly cypress, miscellaneous shrubs, and Miscanthus (stem).

### UAV multispectral imagery collection

A commercial DJI Phantom 4 multispectral UAV (DJI, Shenzhen, China), which is equipped with one Red–Green–Blue (RGB) sensor and five multispectral sensors with each of 2.08 effective megapixels, was used for capturing images of the study area. The UAV has a real-time kinematics (RTK) positioning system based on satellite navigation, which reduces the error of satellite-based position data to the centimeter level. The information about the five bands of the UAV camera is shown in Table 1. The weight, image resolution, and sensor size of the UAV multispectral camera are 1487 g, 1600 × 1300 pixels, and 4.87 × 3.96 mm, respectively. More parameters of the camera are shown in Table 2. Since the camera collects RGB and five spectral images through the sunlight sensor, it records the illuminance information of each image, which facilitates the post-calibration of multispectral images. Additionally, a control panel built into the UAV can be used to automatically adjust the radiometric reflectance calibration and directly acquire reflectance spectral data. Online access to the details of DJI Phantom 4 multispectral UAV is https://www.dji.com/p4-multispectral/specs.

**Table 1:** Spectral parameters of the multispectral camera of the DJI multispectral UAV

Band name	Center wavelength (nm)	Bandwidth (nm)
Red	650	16
Green	560	16
Blue	450	16
NIR	840	26
Red edge	730	16

**Table 2:** Specification of DJI multispectral UAV camera

Criteria	Lens
FOV	62.7°
Focal length	5.74 mm (35 mm format equivalent: 40 mm) infinite fixed focal length
Aperture	f/2.2
ISO range for color sensors	200–800
Monochromatic sensor gain	1–8 times
Electronic global shutter	1/100–1/20000S (visible light imaging)
	1/100–1/10000S (multispectral Imaging)

To ensure consistent environmental conditions such as weather, light, wind direction, and wind speed, we chose to capture UAV images at different altitudes on the same day. The UAV image data acquisition was conducted on a clear and windless day at the times from 12:00 to 2:00 pm on January 13, 2022. The higher the flight altitude, the larger the flight coverage area. The image overlaps the line by 80%. The flight altitudes were set at 100 m (flight surface: 6.01 ha), 200 m (flight surface: 14.01 ha), and 400 m (flight surface: 40.63 ha) above the ground, respectively. The total flight mission takes about two hours to complete.

### UAV image data acquisition

Georeferencing of raw images is achieved through RTK systems. The camera parameters and POS parameters corresponding to each aerial image are obtained according to the calibration results of the UAV camera, the airborne Differential Global Positioning System (DGPS), and the flight controller. Parameters include 178 horizontal and vertical errors less than 0.1 m [35]. Pix4D mapper software (version 4.2.27, Pix4D SA, Switzerland) was used to stitch the complete raw images generated for georeferenced spectral reflectance and VIs mosaic calibration. Aerial triangulation was the initial step in the UAV photogrammetry workflow and could be used to determine the individual orientations of each diorama of the photogrammetry block. The SFM and Multi-View Stereo (MVS) algorithms were performed by the Pix4D mapper, performing bundle adjustment. We oriented an unlimited number of images across the block by bundling adjustments and multiple ground control points (GCPs). The GCPs for each flight has 5 points, one in each of the four corners of the flight area and one in the middle of the area. Aerial triangulation of each sensor also considered its specific lens distortion to determine the position and orientation of each sensor. Combining the above data, a dense point cloud for multi-view stereo matching could be formed, to achieve the purpose of surface reconstruction, and generate orthomosaics images [36].

Next, high-resolution GeoTIFF images including reflectance and VIs were generated, at the same location with different flight altitudes. GeoTIFF images were further processed in ENVI 5.3 (Esri Inc., Redlands, USA) to define regions of interest (ROI). The average reflection spectrum values of objects within the ROI range were taken as the reflection spectrum of the sampling point and the multispectral data corresponding to various ground objects at the sampling point were obtained.

## Methods

### Modeling methods

For karst vegetation coverage detection, we focused on comparing the efficiency of four models: RF, SVM, GBM, and DL.

#### RF

RF is an ensemble classifier that generates multiple decision trees using randomly selected training samples and subsets of variables [37]. This classifier has become popular in the remote sensing community due to its classification accuracy [38, 39]. Not only can RF process and multicollinear high-dimensional data successfully, but it is also fast and insensitive to overfitting, and it is sensitive to sampling design [40, 41]. The variable importance measure provided by RF has been widely used in different scenarios, such as selecting the best variable to classify a specific target class [42].

#### SVM

SVM is a supervised learning model with associated learning algorithms for classification, regression analysis, and outlier detection of data [43]. In addition to reducing the complexity of the approximation function while ensuring the accuracy of data approximation, the SVM algorithm also has a lot of advantages in solving large samples and high-dimensional space problems [44]. At present, it has been successfully applied to spectral analysis research.

#### GBM

To solve the problem that it is not easy to optimize each step of the general loss function, Friedman [45] proposed the gradient boosting machine algorithm, whose idea is borrowed from the gradient descent method. The basic principle of GBM is to train the newly added weak classifier according to the negative gradient information of the loss function of the current model and then combine the trained weak classifier into the existing model in the form of accumulation [46].

#### DL

A DL architecture is a multilayer stack of simple modules that attempts to learn deep features of input data hierarchically through very deep neural networks, many of which compute nonlinear input–output mappings [47]. With multiple layers of nonlinearity in DL, a system can implement extremely complex functions of its input that are sensitive to minute details and insensitive to irrelevant changes in the background and surrounding objects [48]. According to the training process, DL is first initialized hierarchically through unsupervised training and then adjusted in a supervised manner. In this scheme, high-level features can be learned from low-level features, and suitable features can eventually be used for classification [49].

### Spectral vegetation indices selection

Spectral methods are considered potential methods for predicting photosynthetic pigment content, leaf spectral properties are used for reflectance spectra (e.g. VIs), and the physiology of individual trees or populations will be estimated at the stand level [50]. However, different VIs may produce different vegetation characteristic reflections and these indicators may also be affected by different vegetation types. To determine the best variables for vegetation coverage detection, 5 spectral values (Red, Green, Blue, NIR, Red edge) and 16 VIs were selected, as shown in Table 3, including NDVI, OSAVI, GNDVI, SAVI, MSAVI, GCI, RECI, LCI, GRVI, MGRVI, RGBVI, NDRE, MACI, ARI, MARI, and VDVI.

**Table 3:** Details of selected vegetation indices tested in this research

Name	Formula	References
NDVI (normalized difference vegetation index)	(NIR − R)/(NIR + R)	[70]
OSAVI (optimized soil adjusted vegetation index)	((NIR − R)(1 + 0.16))/(NIR + R + 0.16)	[71]
GNDVI (green normalized difference vegetation index)	(NIR − G)/(NIR + G)	[72]
SAVI (soil adjusted vegetation index)	((NIR − R)(1 + 0.5))/(NIR + R + 0.5)	[73]
MSAVI (modified soil adjusted vegetation index)	(2NIR + 1 − √((2*NIR + 1)^2^–8*(NIR − R)))/2	[74]
GCI (green chlorophyll Index)	NIR/G-1	[75]
RECI (red edge chlorophyll index)	NIR/E-1	[75]
LCI (leaf chlorophyll index)	(NIR − E)/(NIR + R)	[76]
GRVI (green red vegetation index)	(G − R)/(G + R)	[77]
MGRVI (modified green red vegetation index)	(G^2^ − R^2^)/(G^2^ + R^2^)	[62]
RGBVI (red green blue vegetation index)	(G^2^ − R × B)/(G^2^ + R × B)	[78]
NDRE (normalized difference red edge index)	(NIR − E)/(NIR + E)	[79]
MACI (modified anthocyanin content index)	NIR/G	[66]
ARI (anthocyanin reflectance index)	G/NIR	[80]
MARI (modified anthocyanin reflectance index)	(G^(−1)^ − E^(−1)^)/NIR	[81]
VDVI (visible-band difference vegetation index)	(2G − R − B)/(2G + R + B)	[82]

NIR, E, R, G, and B represent the near-infrared, red edge, red, green, and blue bands, respectively

The NDVI is the most commonly used indicator of vegetation greenness/vitality, showing strong correlations with Leaf Area Index (LAI) and green biomass, providing information for estimating Net Primary Production and enabling us to distinguish vegetation from non-vegetation [51]. The OSAVI can be used to reduce the effect of soil background on sparse and dry vegetation [52]. The GNDVI is highly sensitive to chlorophyll and reduces non-photosynthetic effects, which can provide valuable information on complex landscapes [53]. The SAVI is more sensitive to vegetation, allowing us to observe areas of potential soil degradation [54]. The MSAVI is commonly used to detect sparsely vegetated areas where soil background influences are important, minimizing external influences and enhancing vegetation signals [55]. A low MSAVI means sparse vegetation, indicating desertification [56]. The GCI is an index for estimating the chlorophyll content of various plant leaves and detecting the physiological state of vegetation, which can be used to evaluate the growth state of vegetation [57]. The RECI constructed from red edge bands is more sensitive than the traditional vegetation index in estimating vegetation biomass [58]. The LCI is a chlorophyll-sensitive vegetation index with a wide range of chlorophyll content and is hardly affected by disturbances caused by scattering [59].

The GRVI is sensitive to leaf color changes (chlorophyll and fall coloration) and can be used to differentiate green vegetation, water, and soil [60]. The GRVI uses the high reflectivity of plants in green (about 540 nm) and the absorption of the red and blue parts of the visible spectrum (400–700 nm) by plant chlorophyll to identify vegetation [61]. The squared band reflectance value helps to amplify the difference between red, green, and blue reflectance [62]. The MGRVI is defined as the normalized difference between squared green reflectance and squared red reflectance, and thus exhibits higher sensitivity in vegetation identification [63]. The RGBVI can be used to extract vegetation cover from drone orthoimages [64]. The NDRE is an important predictor of canopy properties and is very sensitive to canopy chlorophyll content [65]. The MACI correlates with anthocyanin content in plant leaves, providing valuable information about the physiological state of plants [66]. The ARI can be used to assess vegetation health [67]. The MARI has the potential to further aid in the classification of senescent vegetation [68]. Since there are significant differences in the absorption efficiency of vegetation to different wavelengths, the VDVI can be used to detect vegetation pixels and effectively enhance vegetation information [69].

### UAV data analysis methods

In this study, 100 m, 200 m, and 400 m data were randomly sampled through the *e1071* package [83], and *tidyverse* package [84] of *R* software. The collected data is divided into the training set and validation set, of which 80% is the training set and the remaining 20% is the validation set [85]. The RF, SVM, GBM, and DL models were established on test data with flight altitudes of 100 m, 200 m, and 400 m, respectively. All modeling was performed in R software, the *randomForest* package [86] was used for RF modeling, the *caret* package [87] was used for SVM modeling and the *h2o* package [88] was used for GBM and DL modeling, respectively.

### Model accuracy verification

After the models were constructed, the confusion matrix for each model was obtained through the *caret* package [87] in R software. A confusion matrix of size *n* × *n* associated with the classifier shows the predicted and actual classifications, where n is the number of distinct classes [89]. The prediction accuracy and classification error can be obtained from this matrix as follows:

1 $${\text{Accuracy }} = \, \left( {{\text{a }} + {\text{ d}}} \right)/\left( {{\text{a }} + {\text{ b }} + {\text{ c }} + {\text{ d}}} \right)$$

2 $${\text{Error }} = \, \left( {{\text{b }} + {\text{ c}}} \right)/\left( {{\text{a }} + {\text{ b }} + {\text{ c }} + {\text{ d}}} \right)$$

where *a* is the number of correct negative predictions, *b* is the number of incorrect positive predictions, *c* is the number of incorrect negative predictions, and d is the number of correct positive predictions. The fitting and predictive ability of each model were evaluated in combination with the overall accuracy. The higher the overall accuracy, the better the model fitting and prediction ability, and the higher the model accuracy.

In addition, the relationship between the overall accuracy, recall and f1 score of the machine learning model to detect karst vegetation should be considered. The parameters recall (*R*), F1-score (F1), and overall accuracy (OA) were used for RF, SVM, GBM, and DL model performance [30]. Overall accuracy is a widely used metric in classification, it expresses the ratio between the model and the total number of predictions on all test sets. For raw samples and predictions, recall and precision are the ratios of correct predictions to the total number of actual or predicted items in the ensemble, respectively. Generally, precision and recall are a contradictory pair of measures, when one is higher, the other tends to be lower. The F1 score is the harmonic mean of precision and recall, with 1 being the best and 0 being the worst [90].

Three quantities from the performance of a classification process in the population of all instances were used to calculate R, F1, and OA: True positives (TP), false positives (FP), true negatives (TN), and false negatives (FN) using below the equations [91]:

3 $${\text{R }} = {\text{ TP}}/\left( {{\text{TP }} + {\text{ FN}}} \right)$$

4 $${\text{F1 }} = {\text{ 2TP}}/\left( {{\text{2TP }} + {\text{ FP }} + {\text{ FN}}} \right)$$

5 $${\text{AA }} = {\text{ TP }} + {\text{ TNTP }} + {\text{ TN }} + {\text{ FP }} + {\text{ FN}}$$

6 $${\text{OA }} = \, \left( {{\text{AA}}_{1} + {\text{ AA}}_{1} + {\text{ AA}} \ldots \, + {\text{ AA}}_{\text{n}} } \right)/{\text{n}}$$

where *P*, AA, and n are the precision, the average accuracy, and the number of the classes [green vegetation (gv), rock (ro), soil (so), and weed (wd)], respectively. The accuracy of a classification process was defined as the portion of true positives and true negatives in all instances.

### Important variable selection

In predictive modeling, the main concern is to identify the most important predictors included in the reduced model. This can be achieved by identifying the best predictors based on statistical characteristics such as importance or accuracy [92]. Using variable selection to develop predictive models can not only reduce the burden of data collection but also improve predictive efficiency in practice. Since many datasets have hundreds or thousands of possible predictors, variable selection is often a necessary part of predictive model development [93]. In this study, we used the *h2o* package [94] in R software to perform significant variable selection on the model with the highest overall accuracy and to determine the best predictors for the model. Two additional files show this in more detail [see Additional files 1-2].

### Best model inversion and determination

To determine the best model for consistency and accuracy across all flight altitudes, we used the best models built from each flight altitude data to validate the prediction accuracy of the remaining data. After determining on which data the best model is based, it is necessary to test the prediction accuracy of the model for the remaining altitude data. The performance of the best model is tested first on the test set and then on the real set (full original images at flight altitudes of 100 m, 200 m, and 400 m). The images of the three flight altitudes predicted based on the best model are compared with the original images of the respective real data. Finally, the model with the best karst vegetation detection accuracy and karst vegetation retrieval performance was determined.

## Results

### Analysis of modeling accuracy of karst vegetation discrimination

**Fig. 2:**
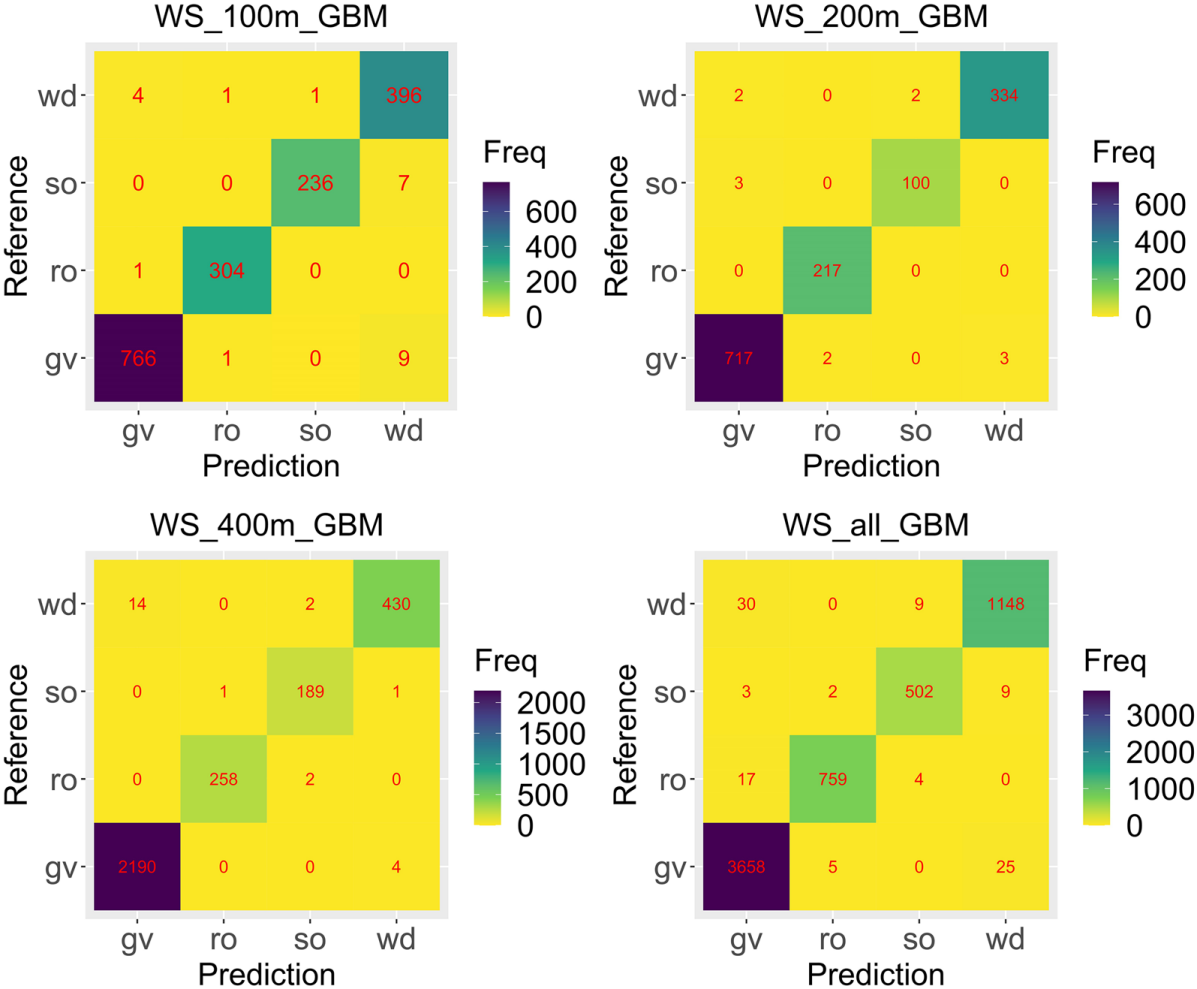
Confusion matrix of best models (GBMs)

The performance of four models using multispectral and VIs at different flight altitudes were shown in Table 4. GBM model yields the best vegetation coverage detection accuracy at all flight altitudes data, with the best accuracies from high to low of 99.11% (200 m), 98.61% (100 m), 98.53% (400 m) and 97.81% (all) respectively. Figure 2 displayed the confusion matrix of each best model (GBM) obtained at different flight altitudes. The highest accuracy of the GBM model was found at 200 m, the model accuracy gradually increases to a certain extent as the flight altitude gradually decreases.

**Table 4:** Parameters of the models at each flight altitude and total

Flight altitude (m)	Model	Classification	*R* (%)	F1 (%)	OA (%)
100	GBM	gv	98.71	99.03	**98.61**
		ro	99.67	99.51	
		so	97.12	98.34	
		wd	98.51	97.30	
	RF	gv	98.84	98.91	98.55
		ro	99.34	99.50	
		so	97.12	98.33	
		wd	98.26	97.29	
	SVM	gv	97.81	98.06	97.51
		ro	98.36	99.17	
		so	97.12	97.72	
		wd	96.52	95.10	
	DL	gv	97.68	97.87	95.83
		ro	98.36	99.17	
		so	83.95	91.07	
		wd	97.51	92.23	
200	GBM	gv	99.31	99.31	**99.11**
		ro	100.00	99.54	
		so	97.09	97.56	
		wd	98.82	98.82	
	RF	gv	99.31	99.03	98.70
		ro	100.00	99.77	
		so	94.17	95.10	
		wd	97.93	98.22	
	SVM	gv	98.89	98.55	97.95
		ro	97.70	98.83	
		so	93.20	92.75	
		wd	97.34	97.48	
	DL	gv	97.92	97.52	92.70
		ro	99.08	94.09	
		so	58.25	73.17	
		wd	97.04	90.36	
400	GBM	gv	98.74	98.58	**98.53**
		ro	99.23	99.42	
		so	90.87	93.33	
		wd	93.89	95.56	
	RF	gv	99.23	98.68	98.45
		ro	98.08	98.65	
		so	88.94	92.04	
		wd	92.36	94.84	
	SVM	gv	98.38	97.65	96.97
		ro	89.23	91.7	
		so	69.71	74.55	
		wd	88.65	93.55	
	DL	gv	96.53	94.13	89.67
		ro	90.77	86.61	
		so	0.48	0.96	
		wd	38.86	55.97	
all	GBM	gv	98.44	98.14	**97.81**
		ro	96.93	97.81	
		so	90.45	93.22	
		wd	95.75	96.27	
	RF	gv	98.60	98.09	97.76
		ro	96.81	97.87	
		so	90.63	93.15	
		wd	95.58	96.06	
	SVM	gv	97.01	96.35	94.68
		ro	93.36	92.71	
		so	65.95	75.39	
		wd	91.83	92.64	
	DL	gv	89.77	93.72	92.45
		ro	93.61	95.01	
		so	81.08	72.00	
		wd	95.41	93.43	

All represents the total including 100 m, 200 m and 400 m data

Bold values represent the highest overall accuracy of the models at each flight altitude

### Mutual validation analysis of optimal models at different flight altitudes

As shown in Table 5, the highest and lowest prediction accuracy rates of 95.66% and 66.15% were found when using the GBM model from all data to detect 100 m data and from 400 m data to detect 200 m data respectively. It can be found that the best model of each flight altitude has the highest accuracy to detect its own training data, but the accuracies show a downward trend to detect other flight altitude data. The above indicates that there is a certain degree of difference in the accuracy of the prediction model when training its own data and the rest of the data.

**Table 5:** Mutual validation parameters of optimal models at different flight altitudes

GBM model flight altitude (m)	Data flight altitude (m)	Classification	*R* (%)	F1 (%)	OA (%)
100	All	gv	74.95	85.03	80.41
		ro	95.53	59.22	
		so	70.63	78.87	
		wd	91.99	90.60	
	200	gv	72.58	83.71	83.19
		ro	99.54	82.92	
		so	82.52	82.52	
		wd	95.56	82.71	
	400	gv	90.35	93.69	**84.96**
		ro	69.23	62.61	
		so	27.40	35.85	
		wd	93.89	76.99	
200	All	gv	98.76	93.53	89.03
		ro	88.76	87.37	
		so	52.43	66.74	
		wd	75.86	82.97	
	100	gv	99.36	96.31	**94.78**
		ro	95.74	97.01	
		so	86.01	92.27	
		wd	90.48	91.39	
	400	gv	97.83	96.72	88.82
		ro	63.08	67.91	
		so	25.48	34.08	
		wd	88.55	80.48	
400	All	gv	87.77	92.53	**80.63**
		ro	91.15	74.71	
		so	64.07	47.77	
		wd	52.21	68.31	
	100	gv	98.44	98.31	80.21
		ro	97.01	69.61	
		so	39.91	49.01	
		wd	52.84	69.14	
	200	gv	78.58	87.04	66.15
		ro	87.96	53.45	
		so	30.21	25.78	
		wd	38.02	55.09	
All	100	gv	97.42	97.92	**95.66**
		ro	96.72	94.10	
		so	85.27	91.17	
		wd	97.26	94.90	
	200	gv	84.30	91.41	89.79
		ro	99.08	85.89	
		so	81.15	78.06	
		wd	97.39	92.82	
	400	gv	93.27	95.58	88.31
		ro	79.69	74.59	
		so	36.89	47.35	
		wd	92.29	78.24	

All represents the total including 100 m, 200 m and 400 m data

Bold represents the highest overall accuracy when the optimal models at different flight altitudes were mutually validated

### Importance analysis of VIs for karst vegetation coverage detection

**Fig. 3:**
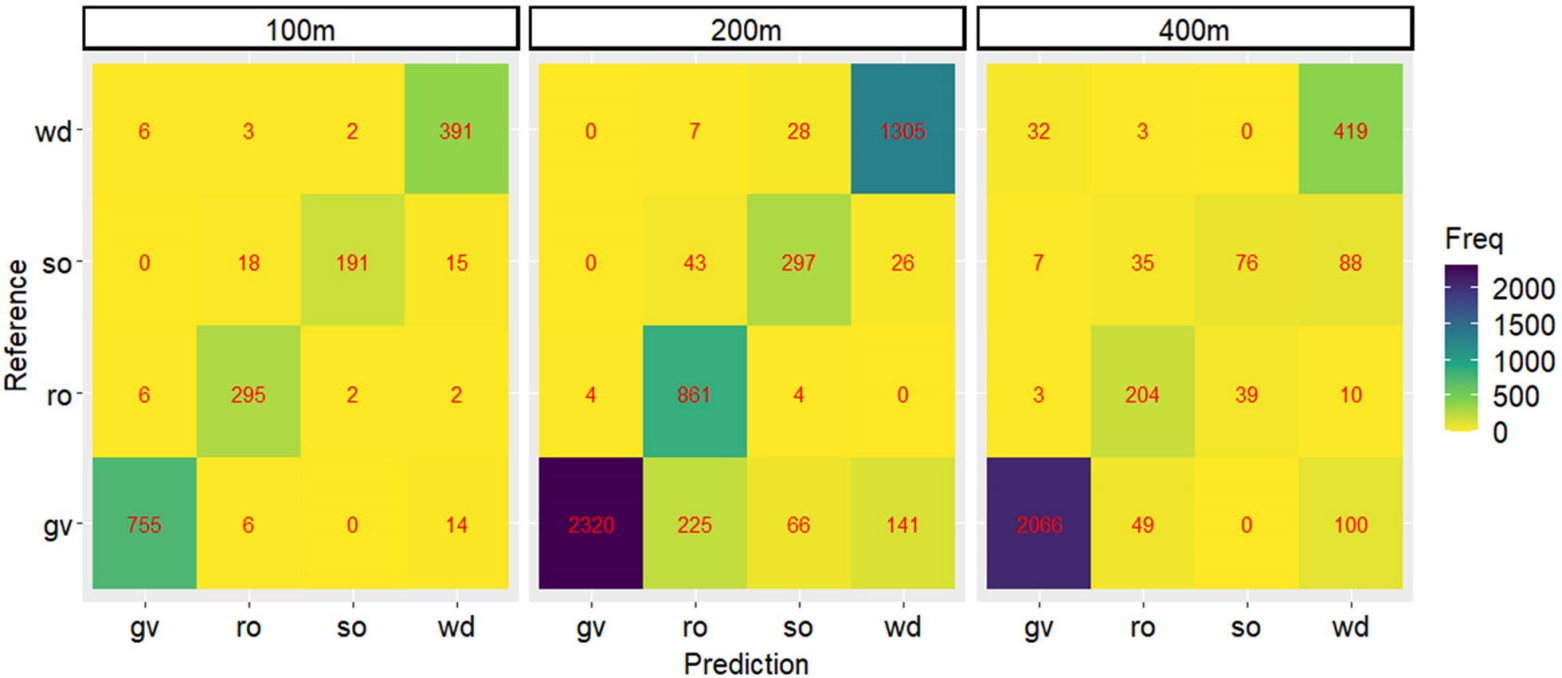
Confusion matrices of GBM models constructed based on overall data for remaining data predictions

It can be known that the GBM model (GBM_all) established based on the overall data works best (Table 5). Figure 3 combined with Table 5 shows that the GBM_all model has the best accuracy of 95.66% when predicting the data with a flight altitude of 100 m, while its accuracy is the worst at 88.31% when predicting the data with a flight altitude of 400 m.

**Fig. 4:**
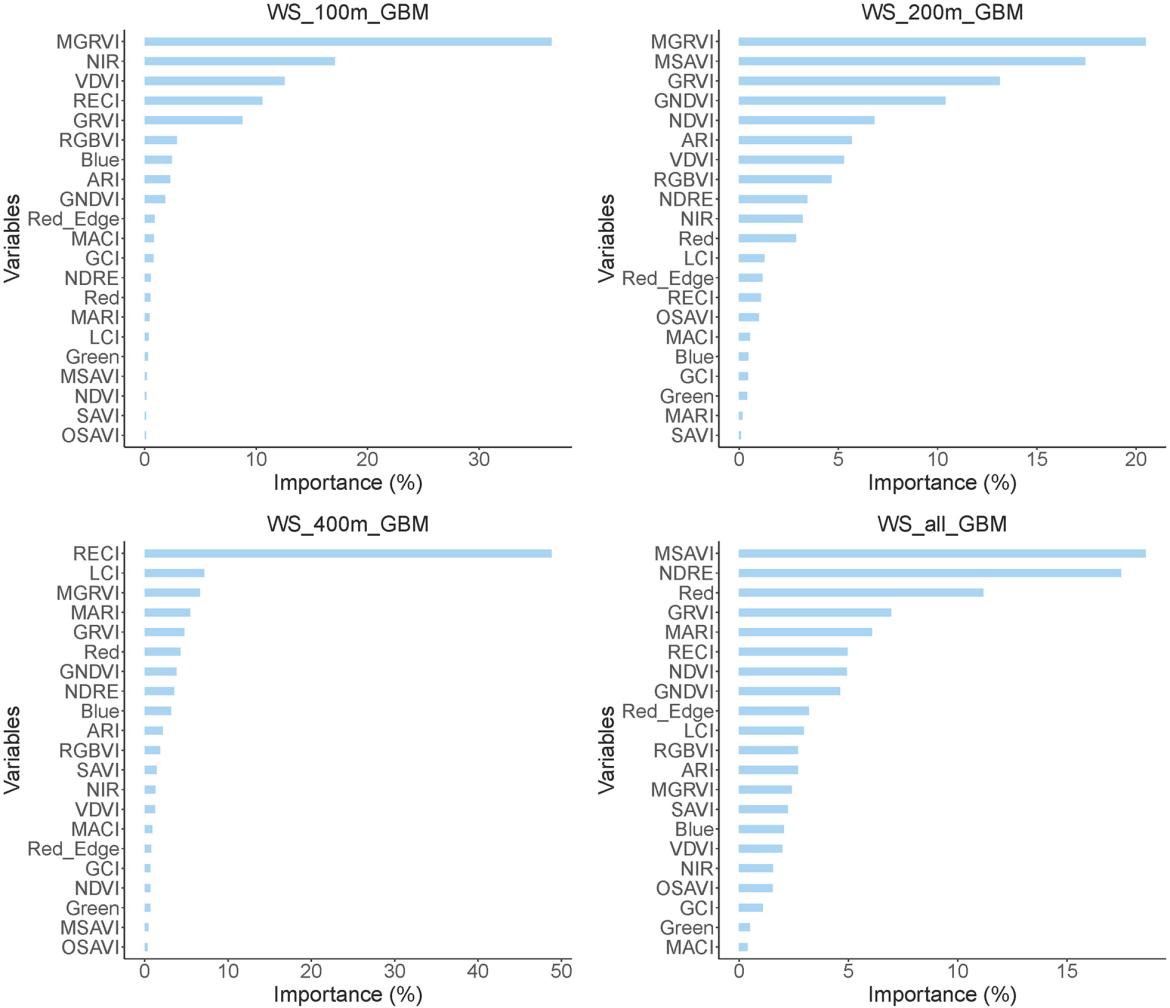
The vegetation indices importance of the best GBM models for each flight altitude

In addition, the vegetation index importance order of the best GBM model at each flight altitude was shown in Fig. 4. The accuracy of GBM models based on different flight altitudes and overall data were significantly correlated with the following vegetation indices: MGRVI (100 m, 200 m), RECI (400 m), MSAVI (all). On contrary, the accuracy of GBM models based on different flight altitudes and overall data was significantly uncorrelated with the following vegetation indices: OSAVI (100 m, 400 m), SAVI (200 m), and MACI (all). The above shows that the prediction models constructed based on data from different flight altitudes have a certain similarity in the distribution of vegetation index importance.

### Inversion of the best model

**Fig. 5:**
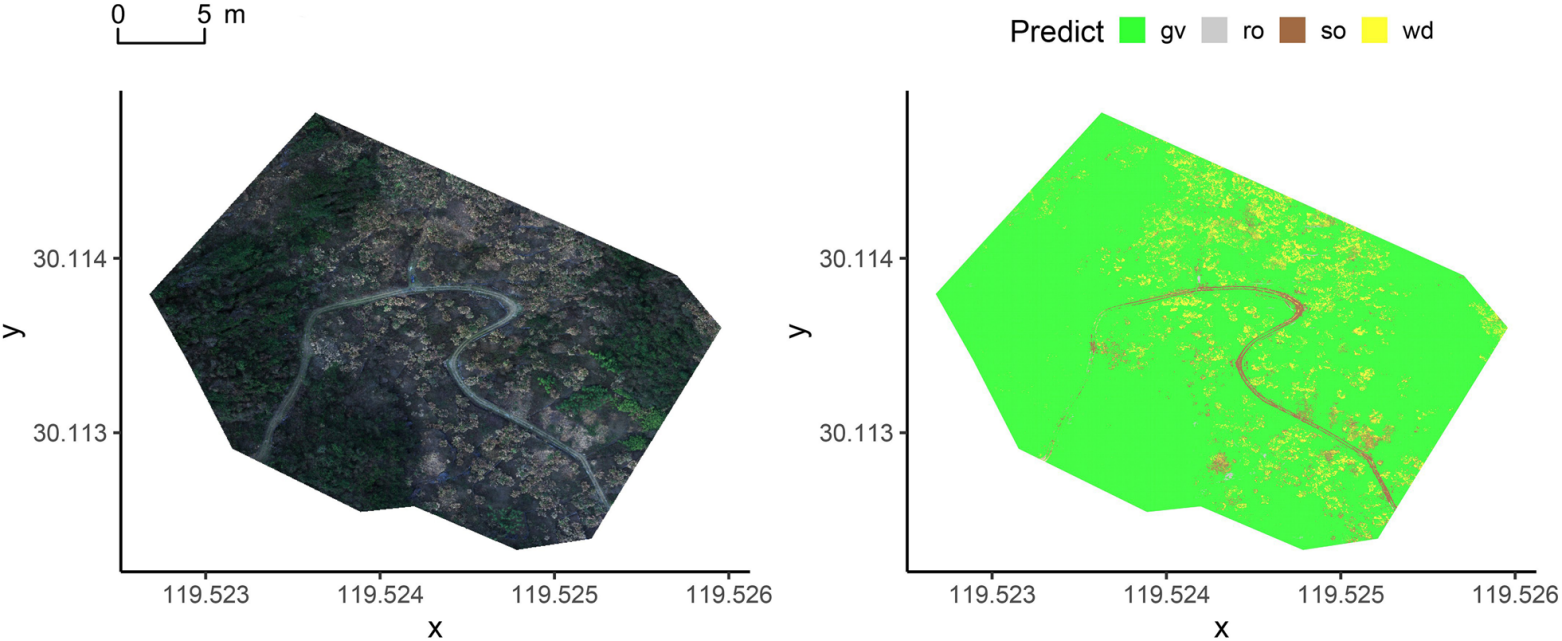
The classification of the best model on the 100 m data. The gv is green vegetation, wd is hay, ro is rock, and so is soil

**Fig. 6:**
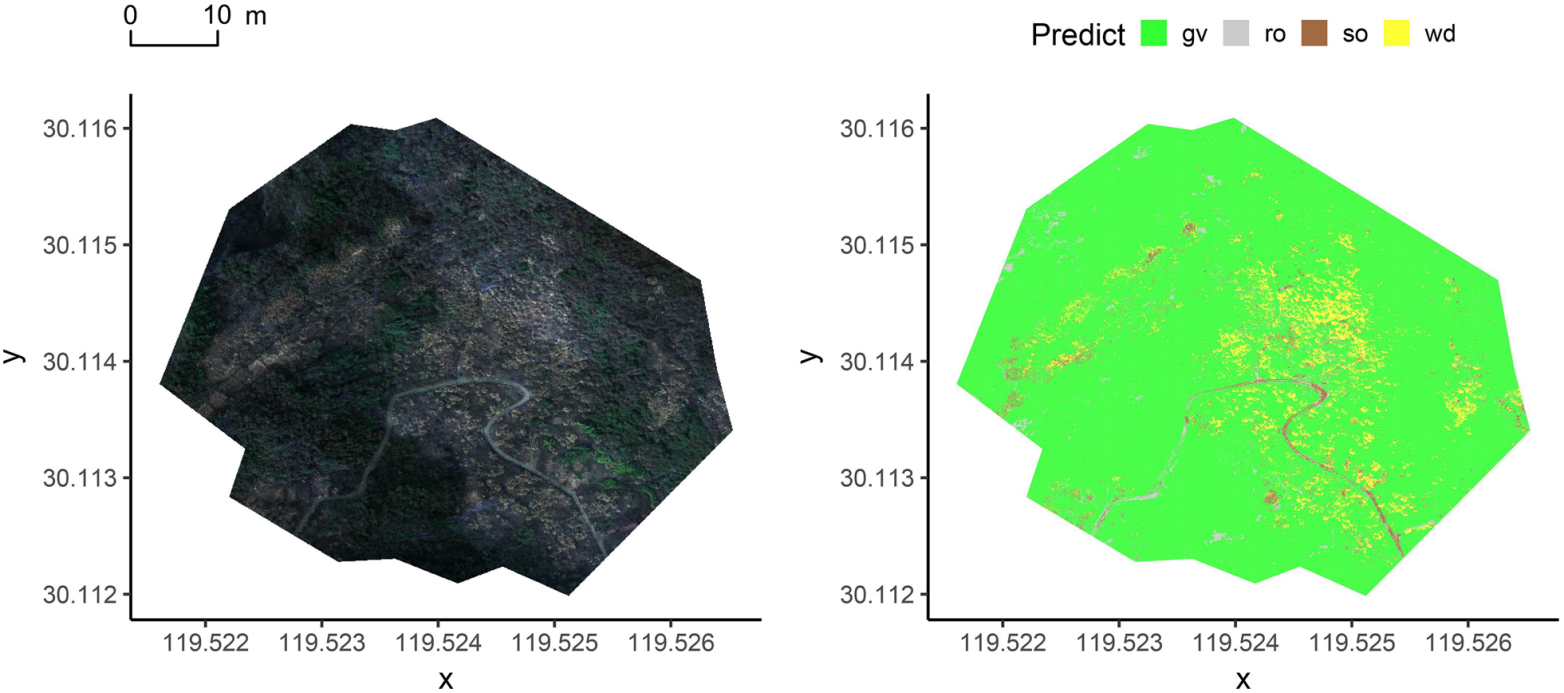
The classification of the best model on the 200 m data. The gv is green vegetation, wd is hay, ro is rock, and so is soil

**Fig. 7:**
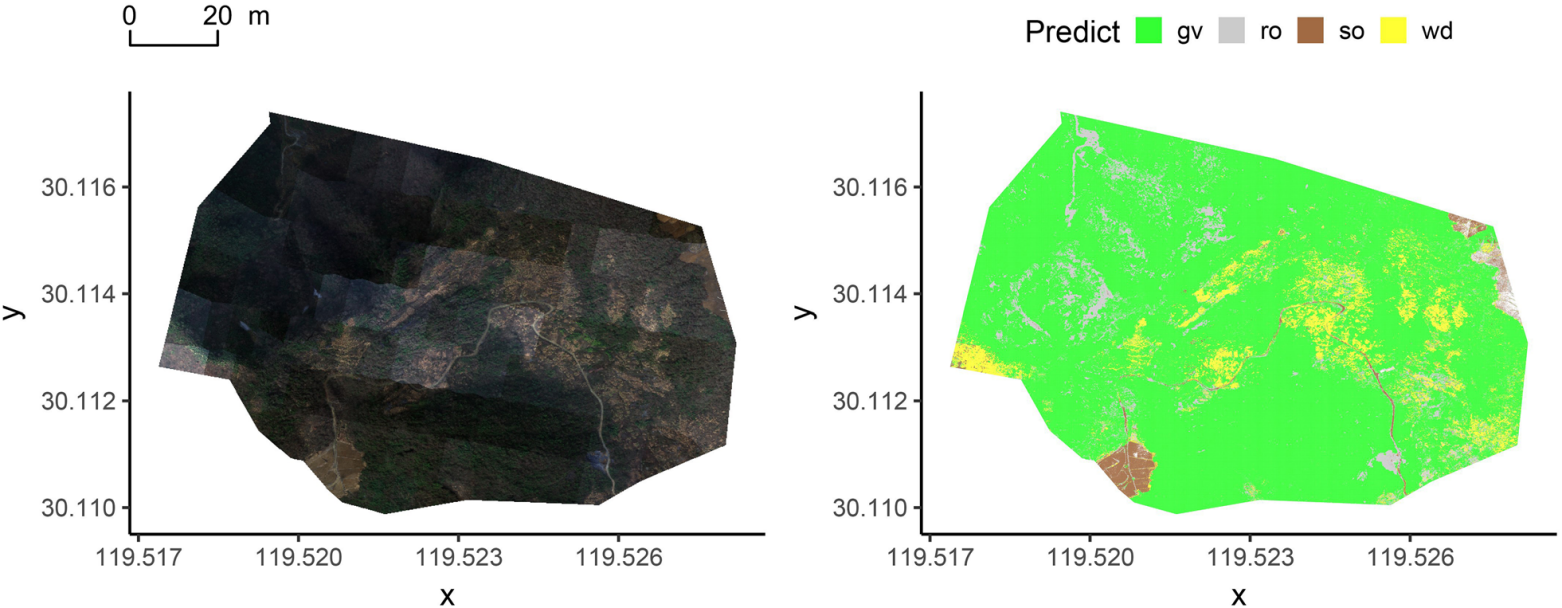
The classification of the best model on the 400 m data. The gv is green vegetation, wd is hay, ro is rock, and so is soil

As shown in Figs. 5, 6 and 7 (the *x* and *y* axes in degrees represent east longitude and north latitude, respectively), the orthomosaics of the three flight altitudes predicted based on the GBM_all model were compared with the original orthomosaics of the respective real data. The orthomosaics for RGB visualization were shown on the left in Figs. 5, 6 and 7. Combined with the method of visual interpretation, the karst green vegetation predicted by the best model was in good agreement with the ground truth, and other land types including hay, rock, and soil were well predicted. It is worth noting that, unlike the southwest karst area where vegetation growth is difficult, the eastern China karst area to which the study area belongs has high vegetation and soil coverage, but low rock exposure.

## Discussion

In the past, Fu et al. [95] combined four single-class SegNet models to classify karst vegetation with an overall accuracy rate of 87.34%. However, the GBM_all model proposed by our study had a higher overall accuracy of 95.66%. Flight altitude determines the final image resolution obtained as well as the effect of topography on radiance (by changing the relative angle between terrain slopes and the UAV) [96]. Many factors affect the setting of the flight altitude, including the weather, temperature of the sampling point, and minor differences in flight altitudes. These factors may complicate the obtained image background and bring challenges to karst vegetation pixel segmentation and model accuracy detection [97]. Larrinaga et al. [98] found that, contrary to expectations, the model fitting accuracy obtained at a higher UAV flight altitude was also higher. But in our research, as the flight altitude gradually decreases, the model accuracy gradually improves to a certain extent.

In our study, the vegetation indices that were significantly correlated and not correlated with the best model (GBM_all) were MSAVI and MACI, respectively, which has already been proven in previous studies. For example, Qi et al. [74] found that MSAVI could increase the dynamic range of vegetation signals, further reduce the influence of soil background, and could be effectively used for vegetation detection in karst areas. Anne et al. [99] found that excessive anthocyanins were mainly related to the juvenile or senescent state of plants, so MACI may not have a significant impact on the detection of karst vegetation coverage.

In addition, in this study, the vegetation indices that were significantly and not significantly correlated with the two flight altitude models were the MGRVI (100 m, 200 m) and the OSAVI (100 m, 400 m). This may be due to differences in lighting during UAV flights [71, 100, 101]. However, Bendig Juliane, Fern et al. [62, 102] found that MGRVI and OSAVI were mainly used for crop identification rather than forestry karst vegetation coverage detection.

Although the method proposed in this study performed well in karst vegetation coverage detection, some aspects could be further improved and explored. First, the resulting images are orthoimages taken by the drone, and vertical cropping of information may cause some loss. To reduce the mutual interference between ground features and improve the accuracy of classification, characteristic parameters can be added to the interpretation process and the layered mask method can be used to select features for each layer image [103]. Therefore, we plan to experiment with layered images in the future to reduce information that can not be displayed due to occlusion. Second, this study only compares four commonly used machine learning methods (RF, SVM, GBM, and DL). Machine learning models have complex parameters and structures [104], so we plan to try a wider variety of machine learning methods in the future. In conclusion, UAV multispectral vegetation indices have high potential in the field of karst vegetation detection and creative results can be achieved when fused with machine learning algorithms.

## Conclusion

In this work, we proposed a GBM_all model based on all flight altitudes image data from UAVs, which could accurately detect karst vegetation coverage with an overall accuracy of up to 95.66%. This study verified the prediction models constructed based on data from different flight altitudes had a certain similarity in the distribution of vegetation index importance. Combined with the method of visual interpretation, we found that the karst green vegetation predicted by the best model was in good agreement with the ground truth, and other land types including hay, rock, and soil were well predicted.

UAV images were beneficial to refine the texture features of the model, improve parameter information, and were more suitable for the detection of karst continuous and complex vegetation. In this study, the combination of UAV images, multispectral vegetation index, and machine learning algorithm has also shown good performance in karst vegetation inversion, providing a reliable and promising method for the identification of vegetation, bare rock, soil in the eastern karst area. In addition, timely and accurate detection of karst vegetation cover will provide important reference information for various forestry management departments in karst areas to reasonably determine karst vegetation restoration plans and evaluate relevant policies.

## Supplementary Information

**Additional file 1.** The vegetation indices importance of the RF models for each flight altitude

**Additional file 2.** The vegetation indices importance of the DL models for each flight altitude

## Data Availability

The Data mentioned in this study are available on request from the corresponding author.
