# Supplementary figures and images for: Karst vegetation coverage detection using UAV multispectral vegetation indices and machine learning algorithm
Source: Plant Methods. 2023 Jan 23;19:7. doi: 10.1186/s13007-023-00982-7 (PMC9869541; doi:10.1186/s13007-023-00982-7)

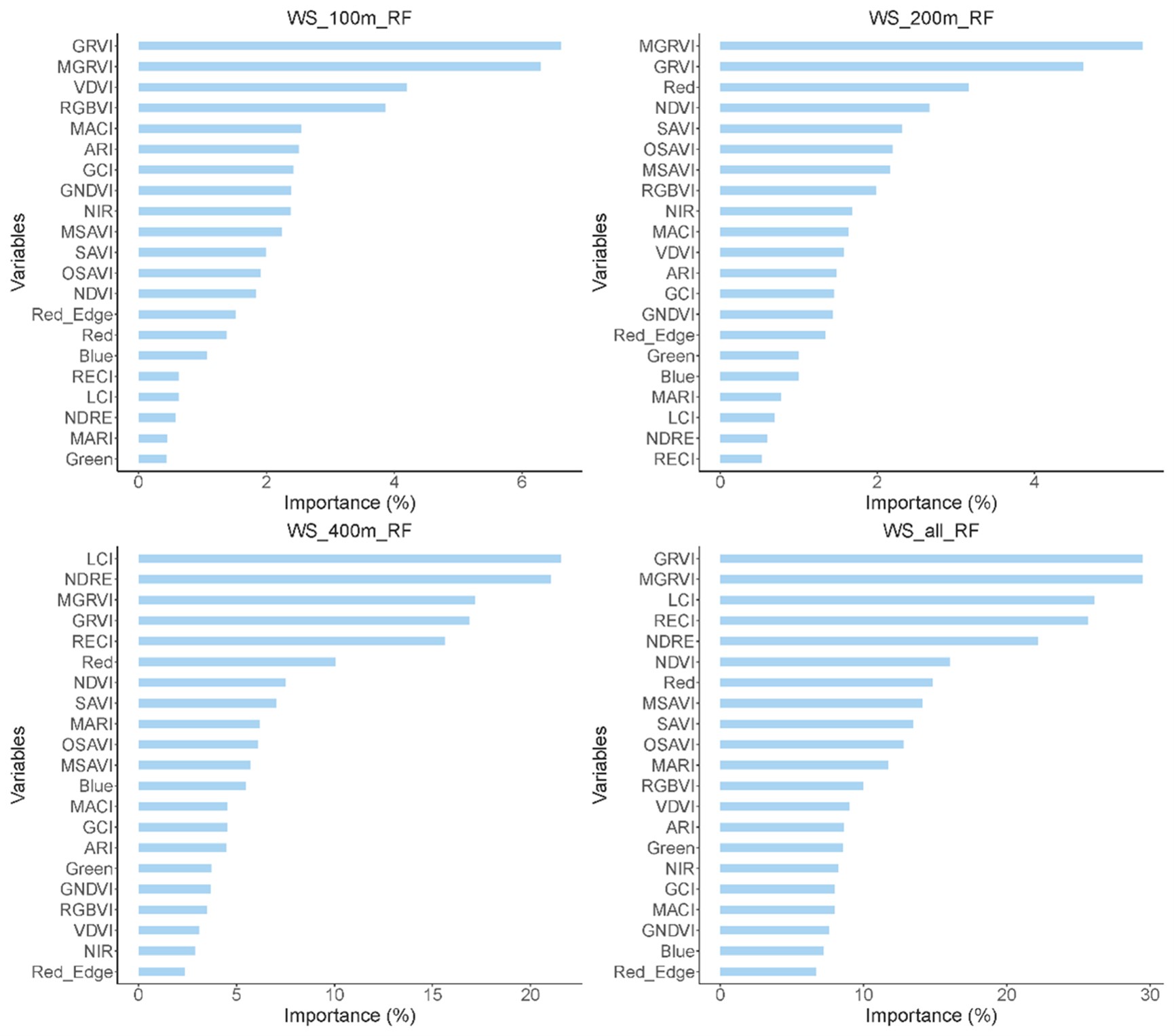

Supplement: Supplementary file 1 — Additional file 1. The vegetation indices importance of the RF models for each flight altitude [file 13007_2023_982_MOESM1_ESM.jpg]

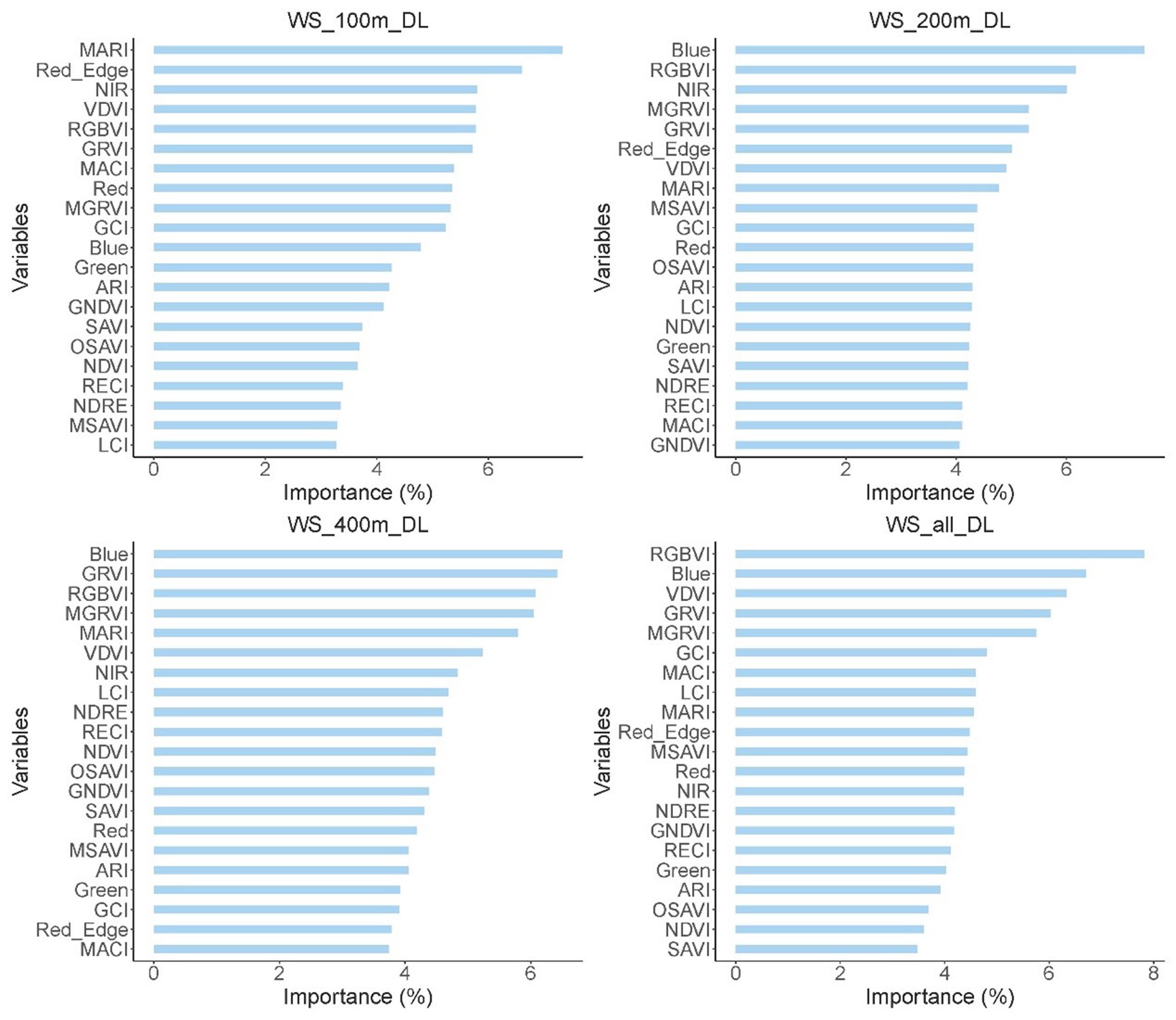

Supplement: Supplementary file 2 — Additional file 2. The vegetation indices importance of the DL models for each flight altitude [file 13007_2023_982_MOESM2_ESM.jpg]
